# Prevalence of clinical manifestations among patients with hypophosphatasia in Central and Eastern European countries

**DOI:** 10.3389/fendo.2026.1892714

**Published:** 2026-07-27

**Authors:** M. Kužma, J. Payer, R. Petrovič, A. Bandura, M. Szegedi, I. Dzivite-Krišane, M. Žerjav Tanšek, J. Haschka, H. Resch, R. Kocijan

**Affiliations:** 1 5th Department of Internal Medicine, Comenius University Faculty of Medicine, University Hospital, Bratislava, Slovakia; 2 Institute of Medical Biology, Genetics, and Clinical Genetics, Comenius University Faculty of Medicine, University Hospital Bratislava, Bratislava, Slovakia; 3 Institute of Genomic Medicine and Rare Disorders, Semmelweis University, Budapest, Hungary; 4 Endocrinology Centre, Children’s University Hospital, Riga, Latvia; 5 Department of Pediatric Endocrinology, Diabetes, and Metabolic Diseases at the Division of Pediatrics, University Medical Centre Ljubljana, Ljublana, Slovenia; 6 Ludwig Boltzmann Institute of Osteology at Hanusch Hospital of OEGK and AUVA Trauma Center Vienna-Meidling, 1st Medical Department, Hanusch Hospital, Vienna Bone and Growth Center, Vienna, Austria; 7 Center of Internal Medicine II, Osteology, Medical Faculty, Sigmund Freud University, Vienna, Austria; 8 Sigmund Freud University, Medical Faculty of Bone Diseases, Vienna, Austria

**Keywords:** alkaline phosphatase activity, ALPL mutations, chronic pain, clinical manifestation, hypophosphatasia

## Abstract

**Mini-abstract:**

Hypophosphatasia (HPP) is a rare systemic disorder with impaired bone mineralization. In 49 Central and Eastern European patients, chronic pain, fractures, deformities, and calcifying periarthritis were frequent. Most had low TN-ALP; only one received asfotase alfa. Findings highlight underdiagnosis, high symptom burden, and need for improved care.

**Introduction:**

Hypophosphatasia (HPP) is a rare, potentially life-threatening, progressive, systemic, inherited metabolic disorder caused by loss-of-function variants in the *ALPL* gene encoding tissue-nonspecific alkaline phosphatase (TNSALP).

HPP is a multi-organ disease, with its hallmark feature impaired bone mineralization.

**Aim:**

The objective of this study was to evaluate the prevalence of HPP in the Central and Eastern European (CEE) region and to assess the frequency of its clinical manifestations.

**Patients and methods:**

A cross-sectional study was conducted on 49 patients with clinically and genetically confirmed HPP. Detailed clinical information was available for 34 patients from five CEE countries.

**Results:**

The analyzed cohort consisted of 14 males and 20 females, with a mean age of 51 years, regardless onset. A total of 33 patients (97%) exhibited TN-ALP levels below the reference range. Only one patient received treatment with asfotase alfa. Chronic musculoskeletal pain was reported by 25 patients (73%), while tooth loss, fractures, and bone deformities were observed in 26%, 44%, and 18% patients, respectively. One patient had a bone mineral density (BMD) in the osteoporotic range, and three patients had a trabecular bone score (TBS) ≤1.23. Other clinical manifestations included calcifying periarthritis (7 patients), seizures (3), nephrocalcinosis (3), hypercalcemia (3), kidney stones (2), ectopic calcifications (1), and pseudogout (1).

**Conclusion:**

This study, based on one of the largest reported cohorts of HPP patients, highlights that chronic pain is the most prevalent symptom. Bone-related complications, such as fractures and deformities, and joint-related conditions, particularly calcifying periarthritis, are also frequent. These findings emphasize the need for greater awareness of HPP, along with dedicated research efforts to enhance patient care and improve access to effective treatments.

## Introduction

Hypophosphatasia (HPP) is a rare, heterogeneous, and potentially fatal inherited disorder characterized by multiple skeletal manifestations, impaired calcium and phosphate metabolism, growth and mobility impairment, premature loss of primary dentition, and, in infants, respiratory difficulties and seizures. It is caused by a mutation in the *ALPL* gene encoding for tissue-nonspecific alkaline phosphatase (TNSALP) (1). To date, over 450 different disease-causing allelic variants of the *ALPL* gene have been identified (2).

The presentation of HPP can be extremely variable both within and between families and can affect both children and adults. Patients at the severe end of the spectrum exhibit profound bone demineralization, pulmonary hypoplasia, respiratory failure, and vitamin B6–responsive seizures. Patients at the mildest end of the disease spectrum may present only with premature tooth loss or periodontal disease (3). The most severe variants affect infants and young children, with symptoms manifesting already *in utero* (1). The main clinical signs relate to defective mineralization of bones and teeth (rickets, osteomalacia, fractures, tooth loss), but in the most severe forms, additional systemic manifestations may be present (seizures, respiratory and renal problems, chronic pain, weakness, etc.) (4). These systemic manifestations may be related to the role of TNSALP in purinergic signaling (via ATP dephosphorylation), which is highly relevant in the central nervous system, bones, and other organs (5).

The basis of the diagnosis of HPP is a persistently low ALP-level in serum (hypophosphatasemia), increased natural substrates of ALP and typical symptoms (e.g.pseudofractures) (3). Moreover, indicators are subsequent elevated concentrations of ALP substrates, i.e., inorganic pyrophosphate (PPi) in blood and urine, pyridoxal-5’-phosphate (PLP, the active form of vitamin B6) in blood, and phosphoethanolamine (PEA) in urine (6, 7). However, genetic testing of the *ALPL* gene must be emphasized as an essential and systematic step in all suspected cases of HPP, since HPP is by definition a genetic disease. Identification of a pathogenic variant confirms the diagnosis and guides clinical management.

Genetic testing of *ALPL* substantially increases diagnostic confidence in suspected HPP, supports counselling and phenotype–genotype interpretation; nevertheless, a negative result does not definitively exclude HPP, as pathogenic variation may reside outside routinely covered regions (e.g., deep intronic, regulatory), or remain unrecognized at the time of testing. Therefore, *ALPL* analysis should be systematically considered whenever biochemical (persistently low ALP with elevated substrates such as PLP/PEA) and clinical findings are concordant. While ALP substrates and advanced biomarkers (e.g., PPi, PLP, PEA; exploratory microRNA assays) may inform diagnosis and disease burden, their routine availability is limited; thus, integration of clinical features with ALP and targeted genetics remains the pragmatic approach in everyday practice.

The diagnosis of HPP is often overlooked in children and especially in adults. One reason for this is that low ALP levels are often ignored, or adequate, age-appropriate reference ranges are missing in laboratory reports (8) Consequently, data from the Global HPP Registry show that the median time between symptom onset and HPP diagnosis is 5.7 years (9). During this time, individuals may suffer from significant multisystem complications of HPP. They may also be misdiagnosed and/or inappropriately treated with medications such as bisphosphonates, which can further worsen the underlying skeletal mineralization defect, increasing the risk of pseudo-fractures (atypical femoral fractures (AFF)) in these patients (10). However, AFFs have been associated with HPP even in patients without bisphosphonate exposure (11). Early and systematic genetic testing is therefore critical to ensure accurate diagnosis and appropriate management of HPP.

Sequencing of the ALPL gene can confirm the diagnosis; however, the presence of a pathogenic *ALPL* gene variant is not observed in all patients with HPP and is not required for diagnosis in patients with repeated low ALP and clinical criteria for HPP (12).

The aim of this registry-based observation study was to evaluate the prevalence HPP-typical skeletal and extraskeletal manifestations in genetically-confirmed HPP patients in the Central and Eastern European (CEE) region.

## Patients and methods

Retrospective, multicentre study across five CEE countries (Slovakia, Austria, Slovenia, Latvia, Hungary). The study was approved by Ethics committee, University Hospital Bratislava, Slovakia, approval no. 94/2024, date 16.10.2024. All procedures were in accordance with the Declaration of Helsinki and local legislation. Data were retrospective and anonymized; [informed consent was given/ethics committee granted exemption from written consent.

Countries were selected based on established collaborations and harmonized diagnostic workflows including centrally coordinated *ALPL* testing. Eligible cases were identified from EMR and reference centre lists; we included only individuals with genetically confirmed HPP and key clinical data available.

**Figure 1:**
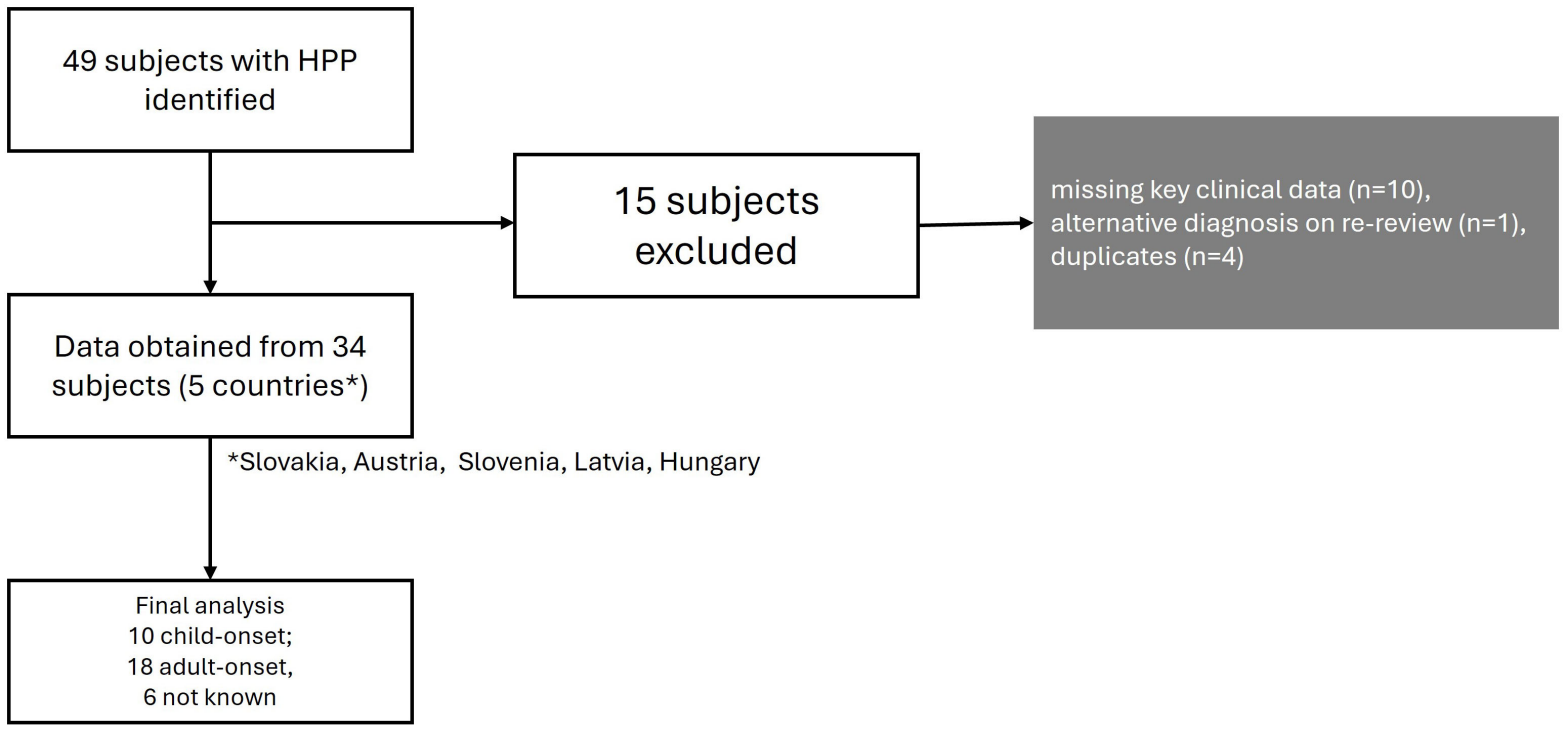
Selection of the study group algorithm.

The study cohort consisted of 49 individuals with confirmed *ALPL* mutations (see selection algorithm in **Figure 1**). Clinical data were available for 34 patients from five countries (Slovakia, Austria, Slovenia, Latvia, and Hungary), who were included in the final analysis.

Patients of all ages were considered, regardless of age at disease onset. Based on documented onset, childhood-onset (≤18 years), adult-onset (>18 years), unknown (insufficient documentation).

### Data collection

Data were collected retrospectively from electronic medical records and physician-reported summaries. The following parameters were systematically recorded:

1. Demographic Data: Age, sex, and country of origin.

2. Clinical Characteristics:

• Bone-related manifestations:

- Fractures/pseudofractures: For each case, the anatomical site (e.g., femur, tibia, metatarsals) and circumstances (e.g., low-energy trauma, spontaneous occurrence, or during normal activity) were documented, along with the time of diagnosis relative to disease course.
- Bone deformities: Defined as clinically or radiologically confirmed alterations in bone shape (e.g., bowing of long bones, varus/valgus deformity, skull shape abnormalities). The location and severity were recorded.
- Chronic musculoskeletal pain: Documented as persistent pain lasting >3 months, unrelated to acute fracture.
- Dental findings: Early tooth loss or dental abnormalities as reported in medical history.
- Joint symptoms: Presence of joint pain, pseudogout, or calcific periarthritis.
- Neuromuscular symptoms: Muscle weakness, hypotonia, or gait disturbances.
- Respiratory complications: Recurrent pneumonia or other respiratory tract infections, particularly in childhood-onset cases.
- Renal involvement: Nephrocalcinosis, kidney stones, or hypercalciuria.

3. Laboratory Parameters:

Serum ALP levels, with HPP defined as ALP below the lower limit of normal (<44 IU/L or <0.58 μkat/L) according to local laboratory standards. Other available biochemical markers (e.g., calcium) were noted when present.

4. Radiological and Bone Density Assessments:

Bone mineral density (BMD) was assessed using dual-energy X-ray absorptiometry (DXA) where available, expressed as Z- or T-scores. Trabecular Bone Score (TBS) was recorded when available, with values <1.23 considered degraded microarchitecture. Radiographs were reviewed for evidence of pseudofractures and deformities.

6. Genetic Testing:

Presence of a known pathogenic variant in the *ALPL* gene were performed in central laboratory at Institute of Medical Biology, Genetics, and Clinical Genetics, Comenius University Faculty of Medicine, University Hospital Bratislava, Slovakia.

### Genetics testing of *ALPL* gene

Genomic DNA was extracted from blood/buccal swabs. Sanger sequencing covered all coding exons (and the first non-coding exon) of *ALPL*; copy number variation (CNV) analysis by MLPA (SALSA P484); next generation sequencing (NGS) custom panel (Agilent SureSelect; Illumina MiniSeq) was used in a subset as per local workflow. Variants were described in HGVS nomenclature and classified per ACMG/AMP (The American College of Medical Genetics and Genomics) into pathogenic/likely pathogenic/VUS; for this analysis we considered pathogenic and likely pathogenic variants as disease-causing.

Metabolite (biomarker) testing: PLP was measured from serum or plasma by high-performance liquid chromatography (HPLC) and PEAby HPLC (DAD detector, derivatised with o-phtalaldehyde -OPA) (13).

7. Associated Treatment Data:

- Use of asfotase alfa (enzyme replacement therapy), vitamin D supplementation, teriparatide, and analgesic therapy.
- The number and type of analgesics used were also recorded.

### Statistical analysis

Statistical analyses were primarily descriptive due to the exploratory nature of the study. Categorical variables are presented as n/N (%) with corresponding 95% confidence intervals (CIs), calculated using the Wilson method. Continuous variables are reported as mean (standard deviation) or median (interquartile range [IQR]), depending on data distribution. Given the limited sample size, formal normality testing was considered of limited interpretability, and non-parametric descriptive measures were preferred where appropriate. Where applicable, comparisons between groups were performed using Fisher’s exact test. A two-sided P value <.05 was considered statistically significant.

## Results

Of the 34 patients included in the final analysis, 20 were female (59%) and 14 male (41%). The mean age at last clinical check-up was 51.1 years (range 27–78 years). In 28.5% (n = 10) of patients HPP manifestation was in childhood, in 51.4% (n = 18) in adulthood, and unknown in 20.0% (n = 6). A known genetic mutation was present in 94.1% of subjects (n = 32) (see **Table 1**). List of mutations are presented in the **Supplementary Table 1**.

**Table 1:** Study group characteristics.

Characteristic	Value
Sex	
Male	14/34 (41.2) [26.4–57.8]
Female	20/34 (58.8) [42.2–73.6]
Age, yr^a^	51.1 (27–78)
Onset of HPP	
Childhood	10/35 (28.6) [16.3–45.1]
Adulthood	18/35 (51.4) [35.6–67.0]
Unknown	7/35 (20.0) [10.0–35.9]
Genetics	
ALPL mutation	32/34 (94.1) [80.9–98.4]
Clinical manifestations	
Dental	9/35 (25.7) [14.2–42.1]
Fractures	15/35 (42.9) [28.0–59.1]
Joint	8/35 (22.9) [12.1–39.0]
Neuromuscular	3/35 (8.6) [3.0–22.4]
Respiratory	10/35 (28.6) [16.3–45.1]
Renal	4/35 (11.4) [4.5–26.0]
Treatment	
Asfotase alfa	1/35 (2.9) [0.5–14.5]
Biochemistry	
Low ALP	33/34 (97.0) [81.4–98.4]
Imaging availability	
DXA available	25/34 (73.5) [56.9–85.4]
TBS available	24/34 (70.6) [53.8–83.2]

^a^Age is presented as median (range). Data are presented as n/N (%) with 95% confidence intervals (Wilson method) unless otherwise indicated. HPP, hypophosphatasia; ALPL, alkaline phosphatase; DXA, dual X-ray absorptiometry; TBS, trabecular bone score.

### Clinical manifestations

**Figure 2:**
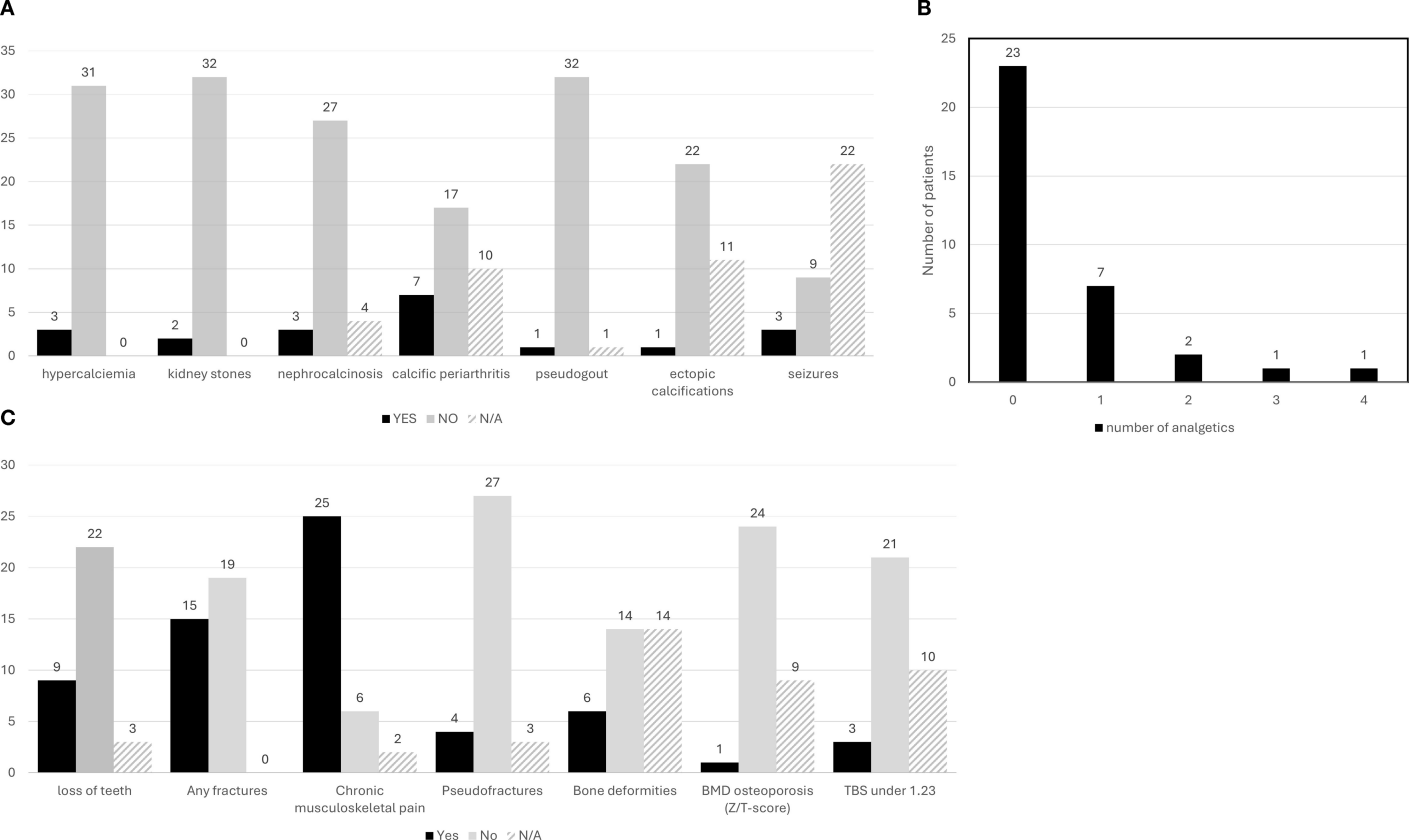
Clinical manifestations and analgesic use among subjects with HPP in CEE countries; **(A)** bone manifestations; **(B)** other manifestations such as rheumatic, kidney and pulmonary; **(C)** analgesic use NA, not available.

Skeletal involvement was highly prevalent among the study population (see **Figure 2A**). Chronic musculoskeletal pain was reported by 73.5% of patients (n = 25), representing the most common clinical feature. Fractures, either traumatic or atraumatic, were documented in 44% (n = 15), while bone deformities were noted in 17% (n = 6). Premature loss of teeth, indicative of early dental involvement, was present in 26.4% of cases (n = 9). Pseudofractures were identified in 11.7% (n = 4). Joint-related symptoms, including arthralgia and suspected pseudogout or calcific periarthritis, occurred in 22.9% (n = 8). **Table 2** is summarizing key manifestations by onset category (child/adult/unknown) visualizing onset-stratified frequencies.

**Table 2:** Key clinical manifestations by onset category (child/adult/unknown) — values shown as n/N (%).

Manifestation	Child onset (n/N, %) [95% CI]	Adult onset (n/N, %) [95% CI]	Unknown onset (n/N, %) [95% CI]	P value*
Chronic pain (any)	6/8 (75.0) [40.9–92.9]	17/18 (94.4) [74.2–99.0]	3/7 (42.9) [15.8–75.0]	.22
Fractures (any)	7/10 (70.0) [39.7–89.2]	8/18 (44.4) [24.6–66.3]	0/7 (0.0) [0.0–35.4]	.25
Pseudofractures	4/9 (44.4) [18.9–73.3]	0/17 (0.0) [0.0–18.4]	0/6 (0.0) [0.0–39.0]	.008
Bone deformities	6/9 (66.7) [35.4–87.9]	0/7 (0.0) [0.0–35.4]	0/5 (0.0) [0.0–43.4]	.011
Premature tooth loss	5/9 (55.6) [26.7–81.1]	4/17 (23.5) [9.6–47.3]	0/6 (0.0) [0.0–39.0]	.19
Calcific periarthritis	1/7 (14.3) [2.6–51.3]	6/13 (46.2) [23.2–70.9]	0/5 (0.0) [0.0–43.4]	.33
Pseudogout	0/10 (0.0) [0.0–27.8]	0/18 (0.0) [0.0–17.6]	1/6 (16.7) [3.0–56.4]	1.00
Respiratory complications	4/10 (40.0) [16.8–68.7]	8/18 (44.4) [24.6–66.3]	1/7 (14.3) [2.6–51.3]	1.00
Renal involvement	1/10 (10.0) [1.8–40.4]	4/18 (22.2) [9.0–45.2]	0/7 (0.0) [0.0–35.4]	.63
Seizures	3/7 (42.9) [15.8–75.0]	0/1 (0.0) [0.0–79.3]	1/5 (20.0) [3.6–62.4]	1.00

N, number of evaluable patients per onset group for a given manifestation; NA, not available.

P values were calculated using Fisher’s exact test (two-sided) for comparison between child-onset and adult-onset groups due to small sample size. Data are presented as n/N (%) with 95% confidence intervals (Wilson method).

Given the age-dependent progression of hypophosphatasia (HPP), an onset-stratified analysis (**Table 2**) revealed distinct phenotypic patterns. Childhood-onset HPP was characterized by a structural skeletal phenotype with frequent fractures (70%), pseudofractures (44%), bone deformities (67%), dental involvement (56%), and seizures (43%). In contrast, adult-onset HPP was dominated by chronic musculoskeletal pain (94%) and showed higher rates of calcific periarthritis (46%) and renal involvement (22%), without major skeletal deformities. Respiratory complications were comparable across groups (~40–44%). These findings support a continuum model of HPP, with early-onset disease reflecting severe mineralization defects and adult-onset disease exhibiting a chronic pain–dominant phenotype with extraskeletal involvement.

DXA report was available in n=25 subjects; n=1 met the criteria for osteoporosis based on BMD. TBS was available in n=24; degraded microarchitecture (≤1.23) in n=3.Extra-skeletal manifestations were also present. Previous, respiratory complications, primarily recurrent lower respiratory tract infections such as pneumonia, were reported in 28.5% (n = 10). Renal involvement, including nephrocalcinosis and nephrolithiasis, was present in 11.4% (n = 4), and hypercalcemia was observed in 8.8% (n = 3). Isolated cases of ectopic calcifications and seizures were also documented, though these were rare (see also **Figure 2B**).

### Laboratory findings

A hallmark biochemical feature of HPP—hypophosphatasemia—was present in the vast majority of patients. Serum alkaline phosphatase (ALP) activity was below the lower limit of normal (<44 IU/L or <0.58 µkat/L) in 97% of patients (n = 33), confirming the biochemical phenotype of the disease. Only 1 asfotase treatment naive patient showed normal levels of ALP.

### Disease associated treatment

Enzyme replacement therapy (ERT) with asfotase alfa was administered in only one patient at the time of assessment. Vitamin D supplementation was used in 38% of cases (n = 13), and teriparatide was prescribed in a single case. Analgetic therapy was common, with 32% (n=11) reporting the use of pain medication. Among these, up to four different analgesics were recorded in some individuals, reflecting the complexity of pain management in this population.

## Discussion

This multicenter observational study provides an overview of the clinical phenotype of hypophosphatasia (HPP) in a Central and Eastern European (CEE) cohort. Our findings underscore the considerable burden of skeletal and extra-skeletal manifestations associated with this rare metabolic disorder. To our knowledge, this is the only dataset focusing specifically on HPP patients from the CEE region.

Consistent with prior studies (9, 14), chronic skeletal pain emerged as the most frequently reported symptom in our cohort, affecting over 70% of patients. The high prevalence of chronic pain is a hallmark of adult-onset and attenuated childhood-onset HPP, and it often significantly impairs quality of life (9, 15–17). The observation that 71% of patients required regular use of analgesics—often multiple concurrent agents—highlights the severity and complexity of pain management in this population. This is in line with existing literature suggesting that chronic pain in HPP is frequently underrecognized and inadequately treated, particularly in adults with a diagnostic delay for many years. Our onset-stratified analysis further supports the concept of age-dependent phenotypic expression in HPP. Childhood-onset disease is primarily characterized by structural skeletal abnormalities reflecting impaired bone mineralization during development, whereas adult-onset HPP presents predominantly with chronic pain and extraskeletal manifestations. This pattern is consistent with previous reports describing HPP as a clinical continuum ranging from severe mineralization defects in early life to a chronic, pain-dominant phenotype in adulthood (18).

Bone fragility, manifesting as fractures, pseudo-fractures or bone deformities was another dominant clinical feature in this cohort. Fractures were reported in over 40% of patients, and more than 17% demonstrated bone deformities. These findings are consistent with the known pathophysiology of HPP, where reduced ALP activity leads to accumulation of pyrophosphate, an inhibitor of hydroxyapatite crystal formation, thereby impairing bone mineralization (19). Notably, pseudofractures, although relatively less frequent (12%), remain a key diagnostic clue, especially in adults with atypical pain and radiological findings. Recently, it has been proposed that patients with hypophosphatasia (HPP) typically do not experience true osteoporotic fractures or generalized osteoporosis, but rather develop pseudofractures, which are characteristic of impaired or defective bone mineralization (20). When true low-trauma fractures are present in HPP subjects, they may indicate concurrent bone loss due to other contributing factors, such as postmenopausal bone loss, prolonged glucocorticoid use, or additional secondary causes of osteoporosis. In our cohort, only a small number of patients exhibited an impaired bone microstructure, reflected by reduced TBS (8%) or met the radiological criteria for osteoporosis, based on BMD measurement by DXA (1 patient).

Dental manifestations, such as premature loss of teeth, were reported in approximately one-quarter of patients, particularly those with childhood-onset disease. This aligns with previous studies describing premature exfoliation of primary teeth as an early and often isolated sign of HPP (12).

Our study also revealed a broad range of extra-skeletal manifestations. Respiratory complications, primarily recurrent pneumonia, were present in nearly 30% of patients, particularly those with earlier disease onset. This likely reflects underlying chest wall deformities, impaired muscle function, or systemic mineralization abnormalities (1). Joint involvement, including calcifying periarthritis and pseudogout, was seen in nearly one-quarter of patients and may relate to the deposition of calcium pyrophosphate crystals due to altered purine metabolism (21, 22). Renal manifestations, including nephrocalcinosis and kidney stones, were reported in over 10% of patients, while hypercalcemia was documented in a similar proportion. These findings further support the systemic nature of HPP and its underlying biochemical imbalance.

Low ALP (ALP <44 IU/L or <0.58 μkat/L) was present in almost all (97%) of patients, reinforcing its diagnostic value. While low ALP activity is a hallmark of HPP (6), it is frequently overlooked in routine clinical evaluations, particularly in adults with non-specific musculoskeletal complaints. In our cohort, the high prevalence of hypophosphatasemia resulted in limited variability in ALP levels, precluding a meaningful correlation analysis between biochemical severity and clinical manifestations. Although low ALP is a hallmark of HPP, it does not consistently correlate with disease severity, reflecting the substantial clinical heterogeneity of the disorder. In our cohort, genetic confirmation of pathogenic *ALPL* mutations was available in 94% of patients. Although not mandatory for diagnosis, genetic testing strengthens diagnostic certainty and allows for better understanding of phenotypic variability. The absence of a detectable *ALPL* variant does not necessarily exclude the diagnosis: the pathogenic change may lie outside routinely covered regions (deep introns/regulatory elements), may be a complex rearrangement or low-level mosaicism – entities that may not be captured by standard panels, exome or Sanger alone; in these situations, WGS, long-read or targeted RNA/functional assays may be beneficial (23). Additionally, variants of uncertain significance (VUS) can be misleading. Due to the reclassification by the ALPL Gene Variant Consortium, numerous VUSs have been reevaluated in recent years, with some being classified as pathogenic (24). In addition, recent studies have identified novel biomarkers (e.g. micro RNA) with potential utility for assessing the extent of multi-organ system involvement in HPP (25). However, miRNAs are currently only available as study tools. Although genetic confirmation was available in most patients, a formal genotype–phenotype correlation analysis was not performed. This was primarily due to the limited sample size and the heterogeneity of ALPL variants identified. Genotype–phenotype relationships in HPP are known to be complex and often inconsistent, with marked intra- and interfamilial variability, limiting the interpretability of such analyses in small cohorts.

Despite the high burden of disease observed, only one patient in our cohort received enzyme replacement therapy (asfotase alfa), the only approved disease-modifying treatment for HPP. There are several reasons for this. Firstly, enzyme replacement therapy is approved for patients with paediatric-onset HPP and bone manifestations. Less than a third of our patients had onset in childhood, and bone manifestations were not evident in all cases. However, it also reflects the limited access to therapy in the CEE region, insufficient awareness among clinicians, and delayed diagnosis. The underuse of targeted treatment underscores the urgent need to improve HPP recognition and care pathways in this part of Europe. There are countries where health insurance providers do not routinely cover the cost of the medication, meaning that patients must rely on special exemptions or individual case assessments. This process is often lengthy and uncertain, making timely and effective treatment difficult to obtain.

This study has several limitations. The retrospective design and relatively small sample size limit the generalizability of the findings and preclude robust inferential statistical analyses. Selection bias may be present, as only patients with available genetic confirmation and clinical data were included. In addition, variability in data collection across participating centers may have influenced the reporting of clinical manifestations. The classification of disease onset may be affected by recall bias, particularly in adult patients with long-standing or nonspecific symptoms.

Potential confounding factors, including comorbidities, age-related bone loss, and prior treatments, could not be systematically controlled and may have influenced the observed phenotype. Furthermore, the high prevalence of hypophosphatasemia limited variability in ALP levels, preventing correlation analyses. Similarly, genotype–phenotype associations could not be reliably assessed due to the heterogeneity of ALPL variants and the limited sample size. These limitations should be considered when interpreting the findings.

In conclusion, based on one of the largest reported cohorts of HPP patients highlights that chronic pain is the most prevalent symptom, often necessitating the use of multiple analgesics. Bone-related complications, such as fractures and deformities, and joint-related conditions, particularly calcifying periarthritis, are also frequent. These findings emphasize the need for greater awareness of HPP, along with dedicated research efforts to enhance patient care and improve access to effective treatments, and the importance of age-specific evaluation and individualized clinical assessment in patients with HPP.

## Data Availability

The raw data supporting the conclusions of this article will be made available by the authors, without undue reservation.

## Supplementary material

Supplementary Table 1 Per-patient genotypes by onset and inferred inheritance in the CEE cohort.
