# Supplementary material for: Prevalence of clinical manifestations among patients with hypophosphatasia in Central and Eastern European countries
Source: Front Endocrinol (Lausanne). 2026 Jul 27;17:1892714. doi: 10.3389/fendo.2026.1892714 (PMC13453749; doi:10.3389/fendo.2026.1892714)
Supplement: Supplementary file 1 [file Table1.docx]

**Suppl.table 1. Per-patient genotypes by onset and inferred inheritance in the CEE cohort**

| **Patient ID** | **Country** | **Onset (Child/Adult/Unknown)** | **Variant 1 c.** | **Variant 1 p.** | **Variant 2 c.** | **Variant 2 p.** | **Zygosity** | **Inferred inheritance (AD/AR)** |
| --- | --- | --- | --- | --- | --- | --- | --- | --- |
| P01 | SLO | childhood | c.1400T>C | p.Met467Thr | c.1114_1115delCT | p.Leu372Aspfs | compound heterozygous (assumed) | AR (inferred) |
| P02 | HUN |  | c.653C>T | p.Ile218Thr | c.1276G>A | p.Gly426Ser | compound heterozygous (assumed) | AR (inferred |
| P03 | LAT | childhood | c.571G>A | p.Glu191Lys | c.335_340dup | p.Gly112_thr | compound heterozygous (assumed) | AR (inferred) |
| P04 | HUN | childhood | c.1374C>A | p.Asp458Glu |  |  | unknown | AD (inferred) |
| P05 | LAT | childhood | c.203C>T | p.Thr68Met |  |  | unknown | AD (inferred) |
| P06 | HUN | adult | c.407G>A | p.Arg136His |  |  | unknown | AD (inferred) |
| P07 | SVK |  | c.299C>T | p.Thr100Met |  |  | unknown | AD (inferred) |
| P08 | SVK |  | c.299C>T | p.Thr100Met |  |  | unknown | AD (inferred) |
| P09 | SVK | childhood | c.299C>T | p.Thr100Met |  |  | unknown | AD (inferred) |
| P10 | SVK |  | c.550C>T | p.Arg184Trp |  |  | unknown | AD (inferred) |
| P11 | SVK |  | c.436G>A |  |  |  | unknown | AD (inferred) |
| P12 | AUT | adult | c.187G>C | p.Ala63Pro |  |  | unknown | AD (inferred) |
| P13 | AUT | adult | c.88C>T | p.Ala30Val |  |  | unknown | AD (inferred) |
| P14 | AUT | adult | c.1310C>T | p.Thr437Met |  |  | unknown | AD (inferred) |
| P15 | AUT | adult | c.984_986delCTT |  |  |  | unknown | AD (inferred) |
| P16 | AUT | adult | c.797_802del |  |  |  | unknown | AD (inferred) |
| P17 | AUT | adult | c.526G>A | p.Ala176Thr |  |  | unknown | AD (inferred) |
| P18 | AUT | adult | c.283G>A | p.Val95Met |  |  | unknown | AD (inferred) |
| P19 | AUT | adult | c.984_986delCTT |  |  |  | heterozygous | AD (inferred) |
| P20 | AUT | childhood |  |  |  |  | unknown | TBD |
| P21 | AUT | adult | c.526G>A | p.Ala176Thr |  |  | unknown | AD (inferred) |
| P22 | AUT | adult | c.648+1G>A |  |  |  | unknown | AD (inferred) |
| P23 | AUT | adult | c.797_802delCCCACT |  |  |  | unknown | AD (inferred) |
| P24 | AUT | adult | c.997+2T>A |  |  |  | unknown | AD (inferred) |
| P25 | AUT | adult | c.984_986delCTT |  |  |  | unknown | AD (inferred) |
| P26 | AUT | adult | c.871G>A | p.Gly291Arg |  |  | unknown | AD (inferred) |
| P27 | AUT | adult | c.997+2T>A |  |  |  | unknown | AD (inferred) |
| P28 | AUT | adult | c.871G>A | p.Gly291Arg |  |  | unknown | AD (inferred) |
| P29 | AUT | childhood | c.526G>A | p.Ala176Thr | c.667C>T | p.Arg223Trp | compound heterozygous (assumed) | AR (inferred) |
| P30 | AUT | adult | c.571G>A | p.E174K | c.1171 | p.R374C | compound heterozygous (assumed) | AR (inferred) |
| P31 | AUT |  | c.304G>A | p.Ala114Thr | c.1310C>T | p.Ala437Val | compound heterozygous (assumed) | AR (inferred) |
| P32 | AUT | childhood | c.648+1G>A |  |  |  | unknown | AD (inferred) |
| P33 | AUT | childhood | c.542C>T | p.Ser181Leu | c.920C>T | p.Pro307Leu | compound heterozygous (assumed) | AR (inferred) |
| P34 | AUT | childhood | c.648+1G>A |  |  |  | unknown | AD (inferred) |

**Legend:** AD = autosomal dominant; AR = autosomal recessive; ACMG class per ACMG/AMP criteria. Inferred inheritance is based on zygosity (heterozygous vs compound heterozygous/homozygous), variant classification and available pedigree data.
